# Invasive Fungal Infections and Targeted Therapies in Hematological Malignancies

**DOI:** 10.3390/jof7121058

**Published:** 2021-12-10

**Authors:** Jessica S. Little, Zoe F. Weiss, Sarah P. Hammond

**Affiliations:** 1 Division of Infectious Diseases, Brigham and Women’s Hospital, Harvard Medical School, Boston, MA 02115, USA; zfweiss@bwh.harvard.edu; 2 Department of Medical Oncology, Dana-Farber Cancer Institute, Boston, MA 02215, USA; shammond@mgh.harvard.edu; 3 Division of Infectious Diseases, Massachusetts General Hospital, Harvard Medical School, Boston, MA 02114, USA; 4 Division of Hematology and Oncology, Massachusetts General Hospital, Harvard Medical School, Boston, MA 02114, USA

**Keywords:** hematological malignancy, fungal infection, targeted therapies, monoclonal antibodies, rituximab, ibrutinib, idelalisib, ruxolitinib, venetoclax, blinatumomab

## Abstract

The use of targeted biologic therapies for hematological malignancies has greatly expanded in recent years. These agents act upon specific molecular pathways in order to target malignant cells but frequently have broader effects involving both innate and adaptive immunity. Patients with hematological malignancies have unique risk factors for infection, including immune dysregulation related to their underlying disease and sequelae of prior treatment regimens. Determining the individual risk of infection related to any novel agent is challenging in this setting. Invasive fungal infections (IFIs) represent one of the most morbid infectious complications observed in hematological malignancy. In recent years, growing evidence suggests that certain small molecule inhibitors, such as BTK inhibitors and PI3K inhibitors, may cause an increased risk of IFI in certain patients. It is imperative to better understand the impact that novel targeted therapies might have on the development of IFIs in this high-risk patient population.

## 1. Introduction

Invasive fungal infections (IFIs) are an important cause of morbidity and mortality in patients with hematological malignancies [1,2]. Defects in innate and cell-mediated immunity related to the underlying disease, cytotoxic chemotherapies, novel targeted immunotherapies, as well as long-term intravenous catheters, and loss of mucosal integrity related to toxic chemoradiation can significantly increase patients’ risk of fungal disease [3,4,5]. Manifestations of fungal infection in patients with hematological malignancies include invasive yeast infections, including invasive candidiasis (IC) including candidemia and deep-seated tissue infection, invasive mold infections (IMI) such as pulmonary and invasive aspergillosis (IA); endemic fungal diseases, such as histoplasmosis or blastomycosis; and classically opportunistic infections (OI) including *Pneumocystis jirovecii* pneumonia (PJP) and cryptococcosis. The epidemiology of IFIs in hematological malignancy has evolved in recent years, driven by the development of novel antineoplastic treatments, changes in practices related to hematopoietic cell transplantation (HCT), and introduction of new antifungal agents for treatment and prophylaxis [1,2,6,7,8,9]. Increased numbers of fluconazole-resistant IC and IMIs present challenges for diagnosis and treatment [2,10]. This shift has occurred in parallel to a rapid expansion of immunotherapeutic and molecular targeted agents [11,12,13]. These agents act on specific molecular pathways theoretically leading to a narrower spectrum of toxicity. However, down-stream sequelae of pathway inhibition may not be fully characterized in early studies and important or unique infectious complications including risks of IFI may not come to light until therapies are applied to broader populations outside of the clinical trial setting. Thus, it is critical to continue to develop a deeper understanding of the unique risks that each agent may have for development of IFIs.

This review describes the reported risk of IFI in patients receiving a variety of targeted agents for hematological malignancies, including monoclonal antibodies; bispecific T-cell engagers (BiTE), such as blinatumomab; tyrosine kinase inhibitors, including phosphoinositide 3-kinase (PI3K) inhibitors, Bruton’s tyrosine kinase (BTK) inhibitors, and janus-associated kinase (JAK) inhibitors; as well as the B-cell lymphoma 2 (BCL-2) inhibitor venetoclax. Given the breadth of this topic and the number of targeted agents now available, the review focuses on the targeted therapies most commonly used in hematological malignancy, where data are present on associated risk or incidence of IFI. For the included therapies, we discuss indications for use, effects on innate and adaptive immunity, reported risk of IFI, and any recommendations regarding antifungal prophylaxis including prophylaxis for PJP. Importantly, as these biologic agents act on specific immune pathways, risk of particular fungal infections may be increased more than others, and it is important to evaluate the reports of invasive yeast infections, invasive mold infections, PJP, and other fungal infections individually. In this way, optimal recommendations may be provided regarding screening, prophylaxis, or preemptive treatment for each agent or class of agents.

## 2. Monoclonal Antibodies

### 2.1. B-cell Depleting Agents

Monoclonal antibodies (MAbs) directed against the B-cell CD20 antigen have revolutionized the treatment of CD20-positive hematological malignancies. In 1997, rituximab was the first anti-CD20 monoclonal antibody approved for the treatment of lymphoid malignancy [14]. Rituximab is a chimeric monoclonal antibody, while newer agents such as obinutuzumab and ofatumumab are humanized and human monoclonal antibodies, respectively [13,15,16]. Rituximab is used to treat non-Hodgkin lymphoma (NHL), Hodgkin lymphoma (HL), chronic lymphocytic leukemia (CLL), and in combination with ibrutinib for Waldenstrom’s macroglobulinemia (WM). Ofatumumab is approved for treatment of CLL, and obinutuzumab is approved for treatment of CLL and follicular lymphoma (fL).

Anti-CD20 MAbs have multiple immune modulating effects that increase risk of infections, as seen in Table 1. Most importantly, Anti-CD20 monoclonal antibodies cause prolonged B-cell depletion through induction of apoptosis, complement-mediated cytotoxicity, and cell-mediated cytotoxicity, leading to deficits in humoral immunity [17]. Decreased B-cell numbers may be seen for at least 6 to 9 months after administration and impaired B-cell maturation may continue for years [13,18]. As plasma cells and B-cell precursors do not express CD20, hypogammaglobulinemia is less frequent, however it can occur following multiple courses of therapy [15]. Though less well defined, downstream effects on cell-mediated immunity may also occur due to the important relationship between B and T-cell function related to antigen presentation and cytokine release [19].

Important effects are also exerted on non-lymphoid cells. Neutropenia is well established as a risk factor of IFI [3,20,21,22]. Severe neutropenia has been reported frequently following rituximab therapy, including late onset neutropenia [23]. Dunleavy et al. reported on 130 previously untreated patients with diffuse large B-cell lymphoma (DLBCL)—76 received chemotherapy (DA-EPOCH) with rituximab and 54 received chemotherapy alone. The incidence of late-onset neutropenia in patients who received rituximab-based therapy was low, but was significantly higher than those who did not receive rituximab (8% vs. 0%; *p* = 0.04) [23]. The median time to onset of late neutropenia was 175 days (range, 77–204 days), with a median nadir of 0.2 × 10^9^/L. Other studies have reported incidence of late neutropenia that ranges from 13% to 24.9% [24,25,26].

Less data regarding neutropenia are available on newer anti-CD20 agents, such as obinutuzumab and ofatumumab. One open-label multi-center phase III trial for ofatumumab as a monotherapy in refractory CLL showed 24% of patients in the ofatumumab group developed neutropenia as compared to 10% in the observation group [27]. Another randomized controlled trial (RCT) compared the efficacy and safety of induction and maintenance therapy with obinutuzumab versus rituximab in patients with fL. Patients received CHOP, CVP, or bendamustine in combination with the anti-CD20 agent for induction, and continued on the anti-CD20 monoclonal antibody as monotherapy for maintenance. Patients in the obinutuzumab group had an increased incidence of grade 3 to 5 neutropenia (45.9% vs. 39.5%) compared to those in the rituximab group, suggesting that obinutuzumab may have an even higher risk of neutropenia than rituximab [28].

Despite the humoral and cell-mediated defects described, the risk of IFI after treatment with anti-CD20 monoclonal antibodies appears to be low as shown in Table 2 [13,15,29]. The exception to this is PJP, where some studies have suggested that there may be an increased risk [13,14]. It remains challenging to determine the impact of individual targeted therapies, including antibodies targeting CD20, on incidence of infectious complications in patients with hematological malignancies given that patients are frequently treated with combination regimens, have often received multiple preceding cytotoxic and targeted treatment regimens, and have significant baseline immune defects related to their underlying disease. The data on any independent increase in risk of infections with the addition of rituximab to existing chemotherapy regimens remain mixed. A systematic review and meta-analysis performed by Lanini et al. reviewed infections in 17 RCTs comparing rituximab with chemotherapy (R-C) to standard chemotherapy (C) for patients with CD20+ malignant lymphomas. Pooled relative risks (RRs) did not indicate increased risk of infections (RR 1.00, CI 0.87–1.14; *p* = 0.943) or death as a consequence of infection (RR 1.60, CI 0.68–3.75; *p* = 0.279) in patients receiving R-C compared to those receiving C, despite increased risk of leukopenia and granulocytopenia in patients receiving R-C [29]. Alternatively, a different meta-analysis of RCTs that included studies evaluating rituximab maintenance therapy versus observation or single-arm phase II trials of rituximab for treatment of lymphoid malignancy showed increased pooled relative risk of infection (RR 2.8, CI 1.3–6.2; *p* = 0.010) for patients receiving rituximab [30]. Fungal infections were not reported individually in these studies. In another review on infectious complications related to MAbs in cancer treatment, no significant increase in the incidence of infections was observed with the addition of rituximab to chemotherapy in eight randomized controlled trials [31].

Novel therapies, such as anti-CD20 monoclonal antibodies, may lead to increased infectious complications in patients with unique immunologic deficits. One randomized trial by Kaplan et al. assessing a unique patient population compared R-CHOP to CHOP for HIV seropositive patients with non-Hodgkin lymphoma. In this study, there were trends toward increased risk of neutropenia and infection with R-CHOP, but most notably there was an increase in deaths related to infections in the R-CHOP group compared to the CHOP group (14% vs. 2%; *p* = 0.035). This was driven principally by patients with low CD4 counts, with 36% of patients with baseline CD4 counts of <50 in the rituximab group that developed fatal infections. Of the 16 infectious deaths, there was one fungal pneumonia, but the majority of infections represented bacterial sepsis. Further reported in the R-CHOP group was a single episode of IC and three episodes of PJP [32]. These frequent and serious complications seem to be unique in the HIV seropositive population.

One small study by Lin et al. looked specifically at fungal infections in 34 patients with DLBCL who received R-CHOP versus 35 patients who received CHOP. They found a significant difference in incidence of fungal infections between the two groups (R-CHOP 41.7%; CHOP 17.1%; *p* = 0.03). All of the cases except for two, in which the organisms were not identified, were due to *Candida* species. No IMIs were reported. The majority of patients (18/20) developed IFI while on chemotherapy. R-CHOP, age > 80 years of age, and bone marrow involvement were risk factors of the development of IFI in univariate analysis. However, while the authors used a definition of IFI that required a positive microbiologic culture for a fungal species, they did not use the European Organization for Research and Treatment of Cancer/Mycoses Study Group (EORTC/MSG) criteria that are commonly used to define IFI, so it is difficult to compare these results to IFI rates in other cohorts with hematological malignancies [33,34].

Non-*Candida* yeast infections have only been reported in isolated cases following treatment with rituximab. In one single-center retrospective study of adults with lymphoproliferative malignancies treated with bendamustine plus rituximab, or bendamustine plus ofatumumab, a single case of cryptococcal infection was reported. Additionally, five patients developed PJP and one patient died of disseminated histoplasmosis 1.5 years after completing rituximab maintenance without additional treatment [35].

The strongest signal of IFI following treatment with rituximab is for *Pneumocystis* pneumonia, though the attributable risk remains small [13,36,37]. Jiang et al. performed a systematic review and meta-analysis of PJP in lymphoma patients treated with rituximab-containing regimens. Seven studies were included in the review—942 patients treated with rituximab and 977 patients treated without rituximab. Patients subjected to rituximab had a higher risk of PJP (RR 3.65, CI 1.65–8.07; *p* = 0.001). Notably, antimicrobial prophylaxis was found to provide a benefit in this setting (RR 0.28, CI 0.09–0.94; *p* = 0.039) [36]. A small case series by Martin-Garrido et al. reviewed 30 cases of PJP in patients who received rituximab, of which 90% were treated for hematologic malignancy. Glucocorticoids were used concomitantly in 73% of patients. Clinical manifestations were severe with 88% developing hypoxemic respiratory failure and 30% mortality [38]. Nonetheless, given the generally weak evidence suggesting increased risk of PJP in patients receiving rituximab-based regimens, the current recommendations are to consider *Pneumocystis* prophylaxis in patients with lymphoma receiving R-CHOP with a 14-day regimen who may be at higher risk or if other risk factors are present. Prophylaxis is recommended for patients receiving fludarabine, cyclophosphamide, and rituximab (FCR) but is otherwise optional in all other cases of rituximab treatment for hematological malignancies [39].

Data on the development of IFI after treatment with novel anti-CD20 monoclonal antibodies, such as ofatumumab and obinutuzumab, are more limited. In a pivotal phase III trial for ofatumumab as maintenance therapy for CLL, infections that represented grade 3 or higher adverse events (AEs) occurred in 13% of patients in the ofatumumab group and 8% of patients in the observation group [27]. In another phase I/II trial by Wierda et al. that evaluated single agent ofatumumab in patients with relapsed or refractory CLL, 189 infectious events were reported among 92 patients. Thirteen infections were fatal, including one related to *Fusarium* infection [40]. Finally, the RESONATE study compared ibrutinib to ofatumumab in R/R CLL with lower rates of infection in the ofatumumab group (54% vs. 70%). There were two cases of bronchopulmonary aspergillosis reported in the ibrutinib group and no fungal infections reported in the group that received ofatumumab [41]. Several other studies have reported isolated cases of IFI related to treatment with ofatumumab, including hepatosplenic candidiasis and other *Candida* infections [42,43].

Obinutuzumab is a third generation anti-CD20 MAb with an engineered Fc region to boost complement-dependent cytotoxicity and antibody-mediated cytotoxicity. In one trial comparing rituximab to obinutuzumab for treatment of fL, the obinutuzumab group had an increased incidence of any grade infection (77.3% vs. 70%) as well as serious infections (18.2% vs. 14.4%) [28]. The GREEN study evaluated the safety of obinutuzumab alone or in combination with chemotherapy for CLL and reported infections in 53.7% of patients (grade ≥ 3 20.1%) [44]. No fungal infections were reported in either of these trials, though a letter to the editor in Annals of Hematology reported two cases of IFI in patients on obinutuzumab monotherapy for CLL, arguing that this may be related to increased potency related to the engineered Fc region. In the first case, the patient developed PJP pneumonia and *Candida krusei* fungemia leading to multiorgan failure and death after being treated with obinutuzumab. In the second case, the patient developed fever unresponsive to broad-spectrum antimicrobials after treatment with obinutuzumab and was ultimately diagnosed with talaromycosis. Both patients were heavily pre-treated with cytotoxic drugs leading to prolonged preceding cytopenias, however both patients also received antifungal therapy during the time of obinutuzumab treatment [45].

### 2.2. Combination Lymphodepleting Agents

Several combination B-cell and T-cell lymphodepleting agents are used for the treatment of hematological malignancy. Alemtuzumab is a humanized IgG1 kappa MAb directed against CD52, a cell surface protein expressed on most normal and malignant B and T lymphocytes [46]. Binding of alemtuzumab to CD52 may cause cell death by complement activation or antibody-dependent cytotoxicity. The CD52 receptor is present on B-cells, T-cells, natural killer (NK) cells, and monocytes. Alemtuzumab leads to prolonged lymphocyte depletion, in particular of CD4+ T-cells that can last up to a year or longer [13,47]. Neutropenia can also occur but typically improves after 2–3 weeks [46]. Alemtuzumab is approved for treatment of CLL.

Alemtuzumab increases the risk of IFI via defects in cell-mediated immunity [47]. In a phase II single-arm multi-center trial evaluating alemtuzumab treatment in patients with CLL who had failed prior fludarabine treatment, multiple episodes of IFI were reported. Fifty-one patients (55%) experienced at least one infection during the study. Fungal infections reported included one case of PJP, three cases of invasive IA including one fatal case, one case of fatal rhinocerebral mucormycosis, one case of IC, and one case of fatal cryptococcal pneumonia [46]. Several other trials were stopped early after internal review, due to high incidence of severe infections in patients who received alemtuzumab [48,49].

Another retrospective study by Martin et al. of infectious complications in patients treated with alemtuzumab for lymphoproliferative disorders in a single institution found that amongst 27 patients, 15 (56%) developed an OI during the study period, including three cases of pulmonary IA, one case of disseminated histoplasmosis, and one case of disseminated cryptococcosis. Nine patients with CLL who went on to receive allogeneic HCT were followed, with 44.4% developing post-transplant OIs, while only 29.6% developed post-transplant OIs in a comparator non-alemtuzumab group. Pulmonary IA was the most common post-transplant OI that was reported. Due to the deficits in cell-mediated immunity and risks of developing PJP, routine prophylaxis is recommended for all patients who receive alemtuzumab [15,50].

Other mixed lymphodepleting monoclonal antibodies include daratumumab and elotuzumab. Daratumumab is a human IgG1 kappa MAb directed against CD38, a cell surface protein that is highly expressed on myeloma cells as well as at low levels on other lymphoid cells, such as regulatory T-cells and B-cells [13,51,52]. It exerts effects via induction of apoptosis, complement-dependent cytotoxicity, and antibody-dependent cytotoxicity. Elotuzumab is a humanized IgG1 MAb directed to signaling lymphocytic activation molecule family member 7 (SLAMF7), a receptor on natural killer cells and plasma cells. Binding of the MAb activates natural killer cells to cause antibody-dependenT-cellular cytotoxicity of SLAMF7-bound plasma cells. Both of these agents are approved for treatment of multiple myeloma [13,53].

There are limited data on infectious complications related to treatment with daratumumab and elotuzumab. In one pivotal study of daratumumab, bortezomib, and dexamethasone for treatment of multiple myeloma, rates of grade 3 or greater infections in the treatment group were similar to the control group (bortezomib and dexamethasone alone) [51]. In another major trial of daratumumab with lenalidomide and dexamethasone for treatment of multiple myeloma, there were slightly more grade 3 or 4 infections in the daratumumab group (28.3% vs. 22.8%). No fungal infections were reported in these trials [54]. Notably another retrospective review of infections in heavily pre-treated myeloma patients receiving daratumumab reported no IFI, suggesting that the risk of IFI may be low in this population [55]. Elotuzumab had similar results in a large phase III trial with infections reported in 81% of patients versus 74% in the control group. After adjustment for drug exposure, rates were equal in the two groups, though the rate of herpes zoster was greater in the elotuzumab group [53]. Again, no fungal infections were reported.

## 3. Bispecific T-Cell Engagers (BiTE)

Blinatumomab is a bispecific anti-CD19 and anti-CD3 human monoclonal antibody. The bispecific antigen binding leads to close interaction between CD3+ T-cells and CD19+ B-cells with resulting T-cell activation and lysis of CD19+ B-cells [11,12,13,16]. It has an indication for treatment of relapsed and refractory B-cell acute lymphoblastic leukemia (ALL) or precursor B-ALL. Blinatumomab leads to sustained B-cell depletion and transient T-cell depletion [11,13]. CD19+ plasmablasts are affected, leading to hypogammaglobulinemia that can last beyond one year [11,15,56]. Neutropenia is also a consequence of blinatumomab with incidence reported from 17–31%, though in one major trial there was a lower rate of neutropenia in the blinatumomab group than the standard chemotherapy group [15,57,58]. Finally, due to T-cell activation, cytokine release syndrome (CRS) and neurotoxicity can occur after administration, frequently requiring administration of corticosteroids or tocilizumab which may represent additional risk factors of infection [13,57].

There appears to be a moderate risk of fungal infections following treatment with blinatumomab, though it remains unclear if this is directly related to the mechanism of blinatumomab, preceding therapies including HCT, or additional immunosuppression related to corticosteroids and tocilizumab in the setting of CRS and neurotoxicity. In a randomized phase III trial of 405 patients with ALL who received blinatumomab or standard chemotherapy, the incidence of grade 3 or higher infection was lower in the blinatumomab group (34.1% vs. 52.3%). However, the rate of IFI was high overall at 10% and IMI appeared more prevalent in the blinatumomab group with six cases of bronchopulmonary aspergillosis and two cases of mucormycosis reported, as shown in Table 3 [57]. The standard chemotherapy group had one case of bronchopulmonary aspergillosis, zero cases of mucormycosis, and one case of *Fusarium* infection. Yeast infections appeared to be more frequent in the chemotherapy group [57]. Notably, following a death from disseminated IFI involving the brain in the phase I TOWER study cohort, antifungal prophylaxis was required for all patients who had undergone prior allogeneic HCT in the subsequent phase II/III trials [12,59]. A small retrospective study of patients receiving blinatumomab with ponatinib for refractory/relapsed ALL also reported one death related to fungal infection [60]. Several other case reports have identified serious IFIs in patients who received blinatumomab [61,62]. While many of these study protocols did not specify whether patients received *Pneumocystis* prophylaxis, it is typically standard of care for patients with ALL, so this should be noted when assessing cases of IFI [39]. Further evidence is needed to clarify the risks of IFI in patients receiving blinatumomab.

## 4. Tyrosine Kinase Inhibitors

### 4.1. Bruton’s Tyrosine Kinase (BTK) Inhibitors

Tyrosine kinases are a family of enzymes that act in intracellular signaling cascades in response to activation by an extracellular messenger [13,16]. Tyrosine kinases may be overexpressed in malignancy and targeted inhibitors are now broadly used for treatment of hematological malignancies [13,72]. Ibrutinib, acalabrutinib, and zanubrutinib are Bruton’s Tyrosine Kinase (BTK) inhibitors, irreversible inhibitors of the BTK protein that act as a signaling molecule in the B-cell receptor pathway, a critical pathway for normal and malignant B-cell maturation and survival [72]. Ibrutinib was the first BTK inhibitor approved and has indications for treatment of CLL, mantle cell lymphoma (MCL), WM, and marginal zone lymphoma (MZL). Acalabrutinib is a more selective second-generation BTK inhibitor approved for MCL and CLL, while zanubrutinib is approved for MCL, WM, and MZL.

BTK protein regulates multiple components of adaptive and innate immunity. BTK is expressed in B-cells, myeloid cells such as macrophages and neutrophils, and platelets [11,13]. Its central role in the B-cell receptor pathway makes it a critical modulator of adaptive immunity [73]. Correlates of BTK inhibition can be derived from the well-known syndrome of X-linked (Bruton) agammaglobulinemia, which is caused by mutation of the BTK gene. Patients experience hypogammaglobulinemia and severe deficits in early B-cell maturation with subsequent risk of life-threatening bacterial infections [72]. BTK is also thought to play a role in innate immunity [73,74]. Recent studies have demonstrated BTK function in Toll-like receptor-mediated recognition of infectious pathogens and recruitment and function of innate immune cells, including phagocytosis by myeloid cells [73,75,76]. Additional immune deficits may be caused by off-target effects on alternative kinases, particularly by the less selective agent ibrutinib [13,72,77].

In terms of characterizing risk of infections, early clinical trials of ibrutinib varied with some reporting a mild increase in risk of infections, including pneumonia, and others reporting no increased risk of infections following treatment with ibrutinib [41,78,79,80,81,82,83]. There was no evidence to suggest an increased risk of IFI specifically until several isolated reports of serious fungal infections, often involving the central nervous system (CNS) in patients receiving ibrutinib were published in 2016 [84]. Compounding those findings, a clinical trial by Lionakis et al. using ibrutinib in combination with chemotherapy for the treatment of primary CNS lymphoma (PCNSL) in 18 patients demonstrated an exceedingly high rate of fungal infections, with seven patients (39%) who developed invasive aspergillosis (five pulmonary; two CNS and pulmonary) and one patient who developed PJP. Two patients who had received systemic corticosteroids prior to treatment for brain edema developed fatal pulmonary and CNS *Aspergillus* infection despite antifungal treatment. To further investigate the high rate of IA, the team developed a BTK knockout model in mice. Mice with the BTK mutation who were infected with *Aspergillus fumigatus* had higher mortality, greater weight loss, and more severe fungal burden assessed by histology, suggesting that BTK may play an important role in the immune response to *Aspergillus* infection [63].

Following the dissemination of this data, multiple other case reports and observational studies were published demonstrating evidence for serious IFI after treatment with ibrutinib. These studies documented cases of disseminated *Fusarium* infection, invasive pulmonary aspergillosis, disseminated cryptococcal infection, and atypical *Pneumocystis* pneumonia [67,82,85,86,87,88,89,90,91,92]. Several systematic reviews reported a significantly elevated risk of infection, with one study describing infectious complications in 56% of patients, with 2% of all patients dying from fatal pneumonia including PJP, histoplasmosis, cryptococcosis, and aspergillosis [91]. Another review that evaluated reports of non-*Aspergillus* IMI in patients who received ibrutinib identified 13 cases in the literature. Nearly all of the patients had underlying CLL, and 10/13 were on ibrutinib monotherapy at the time of diagnosis. Seven patients (54%) had no neutropenia documented—one of the key risk factors of IMI historically. The majority of infections were caused by *Mucorales* spp. and five patients (38.5%) died as a result of the IMI [77]. Observational studies also identified a moderate to high incidence of IFI after treatment with ibrutinib, with infections typically occurring in the first 6 months after initiation of treatment. A multicenter study by Ghez et al. identified 33 patients treated with ibrutinib who developed IFI. The majority (82%) developed IA and 40% had CNS involvement, suggesting a possible increased risk of cerebral involvement in particular [65]. Other cases of IFI identified in this series included disseminated cryptococcosis, mucormycosis, or PJP [65]. Varughese et al. analyzed findings in 378 patients with lymphoid malignancy who received ibrutinib. Serious infections developed in 11.4% of patients with 16 IFIs (37.2%) observed. Risk factors of the development of IFI in univariate analysis included ≥ 3 prior treatment regimens, and receipt of corticosteroids at any time during ibrutinib therapy [64]. One retrospective study following 566 patients at a single center observed a 4.7% 5-year cumulative incidence of OIs, the majority of which were IFIs, though no OIs were observed in patients receiving ibrutinib as first-line treatment [66]. While further clarity is needed, some studies suggest that IFIs may be more frequent for particular underlying diseases, including CLL [77,93].

More limited data on infectious complications are available for newer agents, such as acalabrutinib or zanubrutinib [12,13,94,95,96]. In a phase III, randomized, open-label non-inferiority trial comparing ibrutinib to acalabrutinib for treatment of CLL, the rate of grade 3 or higher infections was comparable between the two groups (30.8% vs. 30.0%). Notably, there were more fungal infections reported in the acalabrutinib group (10; 5 PJP and 5 aspergillosis) compared to the ibrutinib group (5; 2 aspergillosis) [97]. In a pooled analysis of safety data from clinical trials including 1040 patients with B-cell malignancies treated with acalabrutinib, infections of any grade were reported in 67%, and the most common serious infection was pneumonia [98]. Sixty-three fungal infections were identified, including three fatal infections. Amongst the cases of IFI classified as serious, four cases were due to *Aspergillus*, one case was due to *Candida*, and one case was due to *Cryptococcus* [98]. One case report described a patient with CLL treated with acalabrutinib and obinutuzumab who developed cerebral aspergillosis [96]. The ASPEN trial, a phase III study comparing ibrutinib and zanubrutinib showed a similar incidence rate of infections in the two groupsm with only 0.1 OIs per 100 person-months in both groups [99]. Importantly, one zanubrutinib patient developed cryptococcal sepsis. Further monitoring is needed to determine any difference in risk of IFI between these three agents.

**Figure 1:**
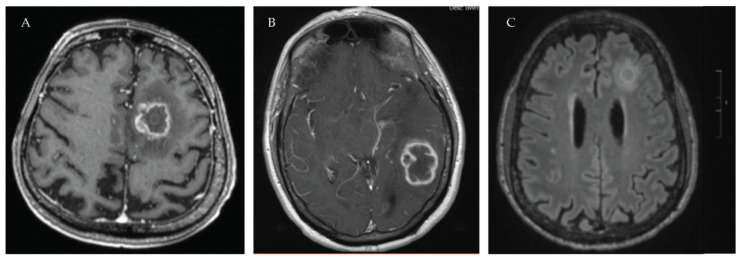
Central nervous system manifestations of IFI in patients receiving BTK inhibitors. IFIs involving the CNS have frequently been reported in patients receiving treatment with BTK inhibitors. The most common and earliest reported CNS infection was IA, by Ruchlemer et al. and Lionakis et al. However, other non-*Aspergillus* mold infections including *Scedosporium*, *Rhizomucor,* and *Lichthemia* sp. have been reported (Anastasopoulo et al.) as well as multiple cases of CNS cryptococcal infections (Chamilos et al.). (**A**) MRI brain of patient with *Scedosporium boydii* infection following treatment with ibrutinib for CLL. (**B**) MRI brain of patient with *Aspergillus fumigatus* brain abscess following treatment with ibrutinib for CLL. (**C**) MRI brain of patient with cryptococcoma following treatment with ibrutinib for Waldenstrom’s macroglobulinemia.

An increased risk of OIs and in particular IMI following treatment with BTK inhibitors was identified primarily through post-marketing surveillance. This risk seems to be most elevated in patients with CLL who have received previous antineoplastic treatment or who receive corticosteroids in conjunction with BTK inhibitor therapy, though it can be difficult to determine the true attributable risk given these other factors [65,66,91,92]. Because of this, guidelines suggest consideration of antifungal prophylaxis in patients treated with BTK inhibitors only if other risk factors are present, such as prolonged neutropenia, old age, high-risk combination therapy, or refractory disease [12,72,100]. Notably, the decision to initiate antifungal prophylaxis or treatment in patients on BTK inhibitors is complicated by significant interactions between BTK inhibitors and triazoles via the cytochrome P450 3A4 (CYP3A4) enzyme [12]. The risk of PJP appears to be low, and *Pneumocystis* prophylaxis is not specifically recommended for patients receiving BTK inhibitors [39,100]. There is also a suggestion that CNS infection may be more frequent after treatment with BTK inhibitors with multiple cases reported. Typical radiographic manifestations of these infections are displayed in Figure 1. Nonetheless, these findings highlight the challenges of identifying serious adverse events related to novel agents in early studies with limited eligibility. In particular, it emphasizes the need for standardized reporting of OIs, including IFI in clinical trials [92].

### 4.2. Phosphoinositide 3-Kinase (PI3K) Inhibitors

Phosphoinositide 3-kinase (PI3K) inhibitors are selective small molecule inhibitors of PI3K, a lipid kinase that acts as a mediator of the B-cell receptor pathway [13,101,102]. PI3K is involved in cell survival and proliferation, and can be hyperactive in B-cell malignancies [11,13,101]. There are multiple isoforms of PI3K, and the δ form which is restricted to hematopoietic cells plays a central role in B-cell development and function, and is the most important isoform involved in the malignant phenotype in CLL [11,16,101,102]. Idelalisib is a reversible inhibitor of PI3K- δ and was the first agent in class approved for treatment of CLL and fL. Duvelisib was later approved for both indications, while copanlisib was approved for relapsed fL alone.

PI3K inhibitors are principally known to inhibit B-cell function via their action in the B-cell receptor pathway. In vitro, PI3K inhibitors have been found to reduce B-cell survival, migration, and antigen-presentation functions, as well as TLR-induced cytokine production, and BCR-mediated B-cell activation and antibody secretion [103,104]. In vivo it can reduce antigen-specific antibody responses and lead to significantly decreased plasma levels of chemokines, including CXCL13, CCL4, and TNFα [104,105,106]. Additionally, idelalisib has been demonstrated to play a role in T-cell function, with one study demonstrating inhibition of T-cell mediated cytokine production, migration, and proliferation [107]. In phase III clinical trials of idelalisib as monotherapy or combination therapy for B-cell malignancies, idelalisib caused grade 3 or higher neutropenia in 27–60% of patients [68,101,102]. Lymphocytosis rather than lymphocytopenia is frequently seen after administration of PI3K inhibitors as monotherapy [101,108]. While patients may suffer from hypogammaglobulinemia driven by their underlying disease, PI3K inhibitors are not thought to confer an additional risk of hypogammaglobulinemia [13,15,101,108].

In terms of risk of infections, initial clinical trials of PI3K inhibitors can be difficult to interpret. While there appears to be an acceptable rate of infectious complications in published trials, multiple phase III trials were halted in 2016 due to high rates of death and serious adverse events for patients receiving idelalisib [109,110,111]. These were largely attributed to OIs, including *Pneumocystis* pneumonia [110]. Amongst published data, one randomized placebo-controlled phase III trial comparing rituximab with idelalisib to rituximab with placebo in patients with CLL reported that incidence of serious pneumonia was similar between the groups (6% vs. 8%), though there were more cases of PJP in the idelalisib group (3% vs. 1%). A second placebo-controlled trial of idelalisib or placebo in combination with bendamustine and rituximab for CLL demonstrated a higher rate of overall infections in the idelalisib group (69% vs. 59%) as well as grade 3 or higher infections (39% vs. 25%). Three patients in the idelalisib group died of pneumonia, including one pulmonary mycosis, three died of sepsis, and two died of septic shock. Importantly, this trial specifically looked at OIs with PJP, which occurred in four patients in the idelalisib group compared to none in the placebo group [68]. Clinical trials for copanlisib also reported several cases of IFIs, including PJP, bronchopulmonary aspergillosis, and cryptococcal meningitis [13,69].

Clinical trials are frequently limited in their assessment of OIs, and these events are rarely reviewed or reported separately. Observational studies can provide additional information by more rigorously reviewing infectious complications in a “real world” population receiving the drug. A study by Marchesini et al. described opportunistic infections in 362 patients with lymphoproliferative disorders treated with ibrutinib or idelalisib. IFI was diagnosed in 13.7% of patients who received ibrutinib and 3.8% of those receiving idelalisib, suggesting that idelalisib may have a lower risk of IFI than ibrutinib [112]. One patient receiving idelalisib died of PJP. In another retrospective study of 462 patients receiving targeted therapies for lymphoid malignancy, the majority of fungal infections was seen in patients receiving ibrutinib, however one case of aspergillosis was reported in a patient receiving idelalisib for treatment of CLL [113]. Additional case reports describe several other serious fungal infections that have arisen in patients receiving idelalisib, including disseminated aspergillosis, concomitant PJP and pulmonary coccidioidomycosis, disseminated cryptococcal infection, and disseminated *Lomentaspora prolificans* infection [114,115,116,117].

While additional studies are needed to clarify the individual risk that PI3K inhibitors may contribute to the development of OIs, early trials demonstrating an increased incidence of PJP in patients not receiving prophylaxis have prompted recommendations that patients receiving PI3K inhibitors should receive *Pneumocystis* prophylaxis [11,72,110,111]. Routine antifungal prophylaxis is not recommended at this time, and CYP3A4 interactions should be considered if prophylaxis or treatment is initiated.

### 4.3. Janus-Associated Kinase (JAK) Inhibitors

Janus-associated kinases (JAKs) are non-receptor tyrosine kinases that regulate signaling of cytokine receptors via the signal transducer and activator of transcription (STAT) pathway [13,16,72]. JAKs are critical to the function of immune and hematopoietic cells. Ruxolitinib inhibits JAK1 and JAK2, which leads to downregulation of T-helper cell type 1 (Th1) responses and cytokines including IL-1, IL6, and TNFα [16]. More recent studies suggest that ruxolitinib may inhibit NK cell and dendritic maturation and function as well [118]. Ruxolitinib is approved for treatment of myelofibrosis, polycythemia vera, and acute and chronic graft versus host disease.

Infections were initially reported as common but largely mild in pivotal trials of ruxolitinib. In the RESPONSE trial, a phase III open-label randomized trial of ruxolitinib versus standard therapy for polycythemia vera, infections occurred in 41.8% of patients in the ruxolitinib group and 36.9% in the standard-therapy group. The rate of grade 3 or 4 infections was slightly higher in the ruxolitinib group (3.6% vs. 2.7%) [119]. In the COMFORT trial, a placebo-controlled phase III trial of ruxolitinib for myelofibrosis, herpes zoster infection was more common in the treatment group but otherwise infectious complications appeared similar between the two groups [120]. No fungal infections were reported. In contrast to these findings, several isolated but serious fungal infections were reported in the literature including cryptococcal pneumonia, PJP, and talaromycosis [121,122,123]. A retrospective study in France identified 4 IFI amongst a cohort of patients receiving ruxolitinib including aspergillosis, cryptococcal pneumonia, PJP, and *Rhizomucor* spp. pulmonary infection [124]

These findings prompted a closer investigation of infections in patients receiving ruxolitinib, and one systematic review by Lussana et al. found an increased risk of *Herpes zoster* infection but did not report any increased risk of fungal infections in patients receiving JAK inhibitors [125]. Further data are needed to understand the immunosuppressive effects of ruxolitinib, however at this point the greatest risks appear to relate to bacterial and viral infections, and in particular *Herpes zoster*, with the risk of fungal infections remaining low [11,13,125]. No specific antifungal or *Pneumocystis* prophylaxis is recommended, unless other indications are present [39,72].

## 5. B-Cell Lymphoma 2 (BCL-2) Inhibitors

Venetoclax, a small molecule inhibitor of the B-cell lymphoma 2 (BCL-2) antiapoptotic protein, is another novel targeted therapy that has seen broad applications in patients with hematological malignancies [11,12]. BCL-2 may be overexpressed by malignant cells in lymphoid malignancies, and inhibition of BCL-2 leads to apoptosis of these cells, representing a promising treatment for lymphoid malignancies [11,126,127]. Venetoclax is an oral agent that is used for the treatment of CLL and in combination with hypomethylating agents for treatment of acute myeloid leukemia (AML). Treatment with venetoclax may actually lead to improvement in T and NK cell immune function related to treatment of underlying CLL [11,128]. Neutropenia is the most notable immune defect reported following treatment with venetoclax. In the MURANO trial, a phase III randomized open-label trial comparing venetoclax plus rituximab with standard chemoimmunotherapy (bendamustine and rituximab) for treatment of CLL, neutropenia was seen in 57.7% of patients in the venetoclax group compared to 38.8% in the standard group, and represented the most common grade 3 or 4 adverse event [127]. Patients with AML may have an even higher risk of neutropenia, given the baseline neutrophil dysfunction that can be caused by the disease. In the VIALE-A trial, a phase III randomized placebo-controlled trial evaluating azacitadine with either venetoclax or placebo for treatment of AML, neutropenia occurred in 42% of patients in the venetoclax group versus 29% that received the placebo [129,130].

The associated risk of infection and in particular fungal infection with venetoclax appears to be low. In the MURANO trial, the incidence of grade 3 or 4 infections was lower in the venetoclax group (17.5% vs. 21.8%) [127]. A comprehensive safety analysis of venetoclax monotherapy for CLL from several phase I/II trials described infections occurring in 72% of patients, with the most common being upper respiratory tract infection (25%) and pneumonia (11%). OIs were reported in 11 patients, including two cases of pulmonary aspergillosis, two cases of PJP, and one case of *Candida* esophagitis, but there were no deaths related to these infections [71]. Patients with AML may have a slightly higher risk of infections, given frequent baseline neutropenia and the effects of cytotoxic chemotherapy potentially exacerbated by venetoclax administration. In the VIALE-A trial, infections of any grade were more common in the venetoclax group (84% vs. 67%) as well as grade 3 or higher infections (64% vs. 51%) [129]. However, no fungal infections were reported. One retrospective study analyzed IFI in a cohort of 119 patients with AML receiving venetoclax with hypomethylating agents [70]. A majority of patients received antifungal prophylaxis, including micafungin (38%), posaconazole (21%), isavuconazole (13%), voriconazole (4%), fluconazole (4%). The overall rate of IFI was low, with fifteen patients (12.6%) who developed IFI including seven cases of aspergillosis, and five cases of mucormycosis. Notably, there were several cases of breakthrough IFI while on prophylaxis. The median time to infection was 72 days. Relapsed or refractory disease and non-response to therapy were risk factors of IFI in the multivariable analysis [70]. Antifungal or *Pneumocystis* prophylaxis is not recommended for patients receiving venetoclax, however patients undergoing initial-induction or salvage-induction chemotherapy for acute leukemia or those with profound, protracted neutropenia are typically recommended to receive antifungal prophylaxis [37,70]. This is particularly important as venetoclax may also have clinically significant interactions with triazoles via CYP3A4 metabolism [131,132].

## 6. Other Novel Agents

There are multiple other novel targeted agents and small molecules with less available evidence regarding increased risk of infections. Antibody-drug conjugates (ADC) are a new class of antineoplastic agents that combine a targeted monoclonal antibody with a linked cytotoxic payload [11]. Inotuzumab ozogamicin is an ADC targeted against CD22, that is used in the treatment of B-ALL [12,16]. In phase III trials, similar rates of neutropenia and lower rates of infection were seen with inotuzumab compared to standard therapy [133]. Gemtuzumab ozogamicin is a similar anti-CD33 ADC used for treatment of AML that also showed no increased incidence of infections in clinical trials [134]. Polatuzumab-vedotin targets CD79b, a B-cell receptor component, and has also not shown a significantly increased rate of infections in trials with combination treatment for DLBCL [135]. Brentuximab vedotin is a fourth in-class ADC that targets CD30 and is used for treatment of HL and certain T-cell lymphomas. One large phase III trial in patients with HL by Moskowitz et al. showed no major increase in infections in the group that received brentuximab, though increased incidence of neutropenia was observed in the brentuximab group [136]. Another trial evaluating brentuximab in combination with chemotherapy for HL showed a slightly increased rate of infections in the brentuximab group that was mitigated by GCSF administration [137]. No fungal infections were reported and the overall risk of IFI with these agents appears to be low.

FMS-like tyrosine kinase 3 (FLT3) inhibitors are another class of selective small molecule inhibitors with widely increased use over recent years. Multiple agents are FDA approved for treatment of FLT3-mutated AML, including midostaurin, gilterinib, and others. Incidence of infection appeared to be similar in a pivotal trial of midostaurin versus placebo in combination with standard chemotherapy for treatment of FLT-3 mutated AML, though in another similar study of gilterinib there were more fatal infectious events in the gilterinib group (28 vs. 7) [138,139]. Continued surveillance is needed to ensure no increased risk of IFI, particularly given the risk factors already present in patients with AML undergoing induction chemotherapy, as well as the potential drug interactions with common antifungal agents used for treatment or prophylaxis [12].

Other agents include BCR-ABL inhibitors used for treatment of CML, ALL, and other hematological malignancies as well as IDH inhibitors used for treatment of AML. These have thus far shown a low risk of infectious complications, particularly IFIs [13,140,141,142].

## 7. Conclusions

Novel targeted therapies are rapidly expanding for treatment of hematological malignancies, and have demonstrated profound impacts on prognosis and treatment-related toxicity. Patients receiving these targeted therapies have myriad underlying risk factors related to their hematological malignancy and previous therapeutic regimens, placing them at risk of infections. This presents significant challenges in determining the individual risk of any particular agent. These novel agents act in signaling pathways leading to downstream effects and unique immunologic sequelae that may not be fully characterized in early studies. Furthermore, targeted therapies are often used in combination with cytotoxic chemotherapy or other novel targeted agents, compounding the impacts on the innate and adaptive immune system.

BTK inhibitors, such as ibrutinib, represent a key example where serious fungal infections were observed frequently outside of the clinical trial setting. This highlights the importance of close monitoring of these agents in a real-world setting, particularly as they are used in an expanded population and in novel combinations. PI3K inhibitors, which act along a similar pathway, also have been reported to have an increased risk of IFI, particularly *Pneumocystis* pneumonia. Blinatumomab and alemtuzumab, monoclonal antibodies that impact B and T-cell function also may increase the risk of developing IFI in patients with hematological malignancy. While thus far there appears to be no increased risk in post-marketing reports, further evidence is needed for many of the newer agents, including the BCL-2 inhibitor venetoclax, the JAK inhibitor ruxolitinib, antibody-drug conjugates, and FLT-3 inhibitors. It is vital to continue to investigate the risk of IFI contributed by these novel agents in populations with a high baseline risk of infectious complications due to immune defects related to the underlying disease, as well as prior treatment regimens. Improved clarity on these risks can optimize prophylactic and preemptive treatment strategies, though it should be noted that significant challenges stem from the major interactions between many of these targeted therapies and traditional antifungal agents such as triazoles.

## Figures and Tables

**Table 1:** Novel targeted therapies: immune sequelae.

Target 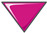	Agents 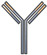	B-Cell Depletion 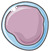	T-Cell Depletion 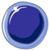	HGG ^1^ 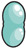	Neutropenia 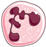
CD20	Rituximab Ofatumumab Obinutuzumab	+++	-	+	++ ^2^
CD52	Alemtuzumab	++	+++	+	+ ^3^
CD38	Daratumumab	+	+	-	+
SLAMF7	Elotuzumab	-	-	-	-
CD19/CD3	Blinatumomab	+++	+	++	++
BTK	Ibrutinib Acalabrutinib Zanubrutinib	++	-	+	+
PI3K	Idelalisib Copanlisib Duvelisib	++	+	-	+
JAK	Ruxolitinib	-	+	-	-
BCL-2	Venetoclax	-	-	-	++

Plus signs indicate relative effect (e.g., mild, moderate, significant). ^1^ Hypogammaglobulinemia. ^2^ Late neutropenia may occur (median time 175 days, Dunleavy et al.). ^3^ Neutropenia typically resolves in 2–4 weeks.

**Table 2:** Novel targeted therapies: risk of invasive fungal infections.

Target	Agents	Risk for IFI	Prophylaxis
BTK	Ibrutinib Acalabrutinib Zanubrutinib	High	● Consider antifungal prophylaxis if other risk factors present. ● PJP prophylaxis if receiving corticosteroid therapy ^1^.
CD52	Alemtuzumab	High	● PJP prophylaxis recommended
PI3K	Idelalisib Copanlisib Duvelisib	Moderate/High	● PJP prophylaxis recommended
CD19/CD3	Blinatumomab	Moderate	● PJP prophylaxis recommended
BCL-2	Venetoclax	Moderate	● PJP prophylaxis if receiving corticosteroid therapy ^1^.
CD20	Rituximab Ofatumumab Obinutuzumab	Moderate/Low	● PJP prophylaxis if receiving corticosteroid therapy ^1^. ● Consider for RCHOP14.
JAK	Ruxolitinib	Moderate/Low	● PJP prophylaxis if receiving corticosteroid therapy ^1^.
SLAMF7	Elotuzumab	Low	Minimal additional risk added
CD38	Daratumumab	Low	Minimal additional risk added
FLT3	Midostaurin Gilterinib	Low	Minimal additional risk added ^2^

^1^ Prednisone equivalent ≥20 mg/day for >4 weeks. ^2^ Given with standard induction therapy during which there is a recommendation for anti-mold prophylaxis.

**Table 3:** Major studies reporting IFI related to use of targeted therapies.

Target	Indication	Reference	Ref No.	Manifestations of IFI
BTK	PCNSL	Lionakis et al.	[63]	IFI incidence 44%; 7 cases of IA including 2 involving CNS; 1 PJP
	CLL NHL	Varughese et al.	[64]	IFI incidence 4.2%; 8 cases of IA; 3 PJP, 1 concurrent IA + PJP, 1 cryptococcosis; 1 *Candida albicans* fungemia
	CLL NHL	Ghez et al.	[65]	33 cases of IFI amongst 16 centers over 4 years; 27 cases IA with 11 involving CNS; 4 cryptococcosis; 1 PJP
	CLL NHL	Rogers et al.	[66]	IFI incidence 3%; 12 cases of IA included 1 involving CNS; 2 mucormycosis; 1 cryptococcosis; 1 blastomycosis; 1 histoplasmosis
	CLL	Frei et al.	[67]	IFI incidence 2.5%; 13 cases of IA; 2 cases invasive candidiasis; 5 cryptococcosis; 1 histoplasmosis; 1 PJP; 1 *Fusarium* infection
PI3K	CLL	Zelenetz et al.	[68]	1 patient died from pulmonary mycosis; 4 cases of PJP
	NHL	Dreyling et al.	[69]	IFI incidence 2%; 1 case of IA; 2 PJP
CD19/CD3	ALL	Kantarjian et al.	[57]	IFI incidence 10% in blinatumomab group; 6 cases of IA; 2 mucormycosis; 1 PJP
BCL-2	AML	Aldoss et al.	[70]	IFI incidence 12.6%; 7 cases of IA; 5 cases of mucormycosis; 2 *Scedosporium*; 1 *Penicillium*
	CLL	Davids et al.	[71]	IFI incidence 2%; 2 cases of IA; 3 cases oral/esophageal candidiasis; 2 PJP

## Abbreviations

IFI	Invasive fungal infections
IC	Invasive candidiasis
IMI	Invasive mold infection
IA	Invasive aspergillosis
OI	Opportunistic infection
PJP	Pneumocystis irovecii pneumonia
HCT	Hematopoetic Cell Transplantation
BiTE	Bispecific T-cell Engagers
BTK	Bruton’s Tyrosine Kinase
PI3K	Phosphoinositide 3-kinase
JAK	Janus-associated Kinase
BCL-2	B-cell lymphoma 2
MAb	Monoclonal antibody
NHL	Non-Hodgkin’s lymphoma
HL	Hodgkin’s lymphoma
CLL	Chronic lymphocytic leukemia
WM	Waldenstrom’s macroglobulinemia
fL	Follicular lymphoma
HGG	Hypogammaglobulinemia
DLBCL	Diffuse large B-cell lymphoma
RCT	Randomized controlled trial
R-C	Rituximab + chemotherapy
C	Standard chemotherapy
RR	Relative risk
AE	Adverse event
EORTC/MSG	European Organization for Research and Treatment of Cancer/Mycoses Study Group
NK	Natural killer cell
SLAMF7	Signaling lymphocytic activation molecule family member 7
ALL	Acute lymphoblastic leukemia
MCL	Mantle cell lymphoma
MZL	Marginal zone lymphoma
CNS	Central nervous system
PCNSL	Primary central nervous system lymphoma
CYP3A4	Cytochrome P450 3A4
AML	Acute myeloid leukemia
ADC	Antibody-drug conjugate
FLT3	FMS-like tyrosine kinase 3
